# Multiple Cerebral Infarctions Complicating Deep Vein Thrombosis Associated With Uterine Adenomyosis: A Case Report and Literature Review

**DOI:** 10.7759/cureus.28061

**Published:** 2022-08-16

**Authors:** Mitsuyoshi Tamura, Akiyuki Uzawa, Yoshihisa Kitayama, Yuji Habu, Satoshi Kuwabara

**Affiliations:** 1 Department of Neurology, Chiba University Graduate School of Medicine, Chiba, JPN; 2 Department of Reproductive Medicine, Chiba University Graduate School of Medicine, Chiba, JPN

**Keywords:** trousseau’s syndrome, cancer antigen 125, deep vein thrombosis, uterine adenomyosis, cerebral infarction

## Abstract

We describe a 46-year-old woman who developed multiple cerebral infarctions in the left middle cerebral artery territory and deep vein thrombosis, presumably related to uterine adenomyosis. Uterine adenomyosis can cause coagulation abnormalities, as observed in Trousseau’s syndrome. Along with previous reports, our case experienced a stroke during menstruation and presented with increased cancer antigen 125 (CA125) levels. A hysterectomy was performed to prevent the recurrence of cerebral infarction. Our case also had complicated deep vein thrombosis, which is also known as a complication of uterine adenomyosis. We consider cerebral infarction and deep vein thrombosis with uterine adenomyosis might be caused by a common mechanism, hypercoagulation. Hysterectomy requires careful discussion before undergoing it because of fertility problems, but it might be the most effective approach for preventing the recurrence of brain infarction derived from adenomyosis and may be effective for both cerebral infarction and deep vein thrombosis.

## Introduction

Uterine adenomyosis, a common disease in which endometrial-like tissue is formed in the myometrium, has been known to cause menstrual pain and menorrhagia, with reports showing a prevalence of approximately 20% among women. Uterine adenomyosis can induce venous thromboembolism in 12.2% of cases [1], during menstruation or hypercoagulation due to tissue factors [2], causing elevated levels of cancer antigen 125 (CA125) [3] associated with menstruation. It has been shown that hypercoagulability associated with uterine adenomyosis can cause cerebral infarction [4], or deep vein thrombosis [1]. Here, we report on a case of cerebral infarction and review the literature on cases of uterine adenomyosis-associated cerebral infarction.

## Case presentation

**Figure 1:**
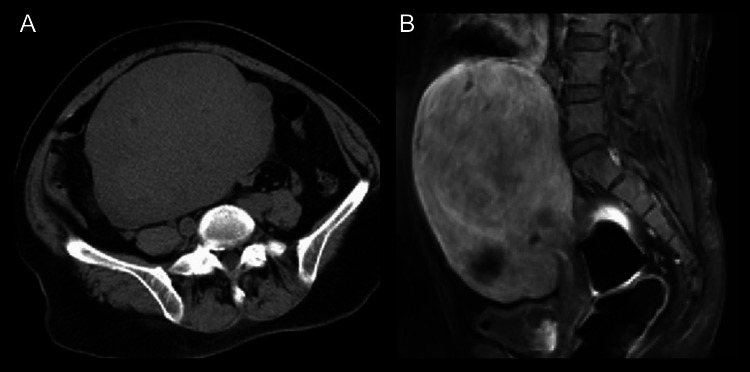
Pelvic tumor Internally uniform pelvic tumor with smooth edges was observed during axial CT (A). T2-weighted magnetic resonance imaging suggested no malignancy (B).

A 46-year-old Japanese woman with no medical history developed acute right hemiplegia and aphasia, for which she was admitted to a nearby hospital. According to the family, she had mild right paralysis a day before being transported to the emergency room. Brain magnetic resonance imaging (MRI) revealed acute cerebral infarctions in the left middle cerebral artery territory. Treatment with aspirin, ozagrel sodium, and heparin (5,000 units per day) was started. Given the presence of a pelvic tumor on computed tomography (CT) (Figure 1A), she was transferred to our gynecological department on day four on suspicion of Trousseau’s syndrome.

**Figure 2:**
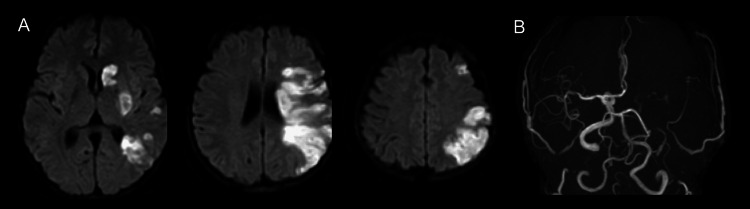
Cerebral infarction Diffusion-weighted imaging revealed a new infarction in the left middle cerebral artery region (A). The lesion included a part of the cortex (especially the parietal lobe). Left internal carotid artery obstruction and the development of collateral circulation were observed (B).

On admission, her blood pressure, pulse, and body temperature were 195/121 mmHg, 75/min, and 37.5°C, respectively. She had severe right hemiplegia with facial involvement and total aphasia, and was awakened but unable to communicate in the language. Blood tests revealed diabetes mellitus, anemia, and elevated D-dimer concentrations. Serum levels of CA125 and carbohydrate antigen 19-9 (CA19-9) were high (Table 1). T2-weighted MRI of the pelvic tumor suggested no malignancy (Figure 1B). Brain MRI revealed a fresh infarction in the left middle cerebral artery region, which included the cortex (Figure 2A), whereas magnetic resonance angiography revealed obstruction at the left internal carotid artery, with the development of collateral circulation (Figure 2B). No arrhythmia, such as atrial fibrillation, was observed on electrocardiography. Transesophageal echocardiography was performed to determine the source of the embolism, although no abnormalities that could cause cerebral infarction were found, such as a patent foramen ovale or intracardiac thrombosis. Contrast-enhanced CT showed deep venous thrombosis in the right lower extremity and a pulmonary embolus. Needle biopsy of the uterus led to the diagnosis of adenomyosis.

**Table 1:** Laboratory investigations PT-INR: prothrombin time-international normalized ratio; APTT: activated partial thromboplastin time; CA125: cancer antigen 125; CA19-9: carbohydrate antigen 19-9.

Tested item	Value	Reference range
Glucose	227 mg/dL	73-109 mg/dL
Hemoglobin A1c	8.6%	4.6-6.2%
Hemoglobin	9.6 g/dL	13.7-16.8 g/dL
Platelet count	404,000/μL	150,000-450,000/μL
PT-INR	0.94	0.8-1.2
APTT ratio	1.0	<1.4
D-dimer	7.4 μg/mL	<0.5 μg/mL
Antithrombin III values	80%	80-130%
Protein C	123%	64-146%
Protein S	55%	56-126%
Antineutrophil cytoplasmic	Negative	Negative
Antiphospholipid antibodies	Negative	Negative
CA125	1477 U/mL	<24.5 U/mL
CA19-9	334.8 U/mL	<36.8 U/mL

Given that brain infarction emerged during menstruation, we considered coagulation disorder caused by adenomyosis to have been the main cause. The gradual progression of symptoms/signs generally suggests atherothrombotic infarction, but we interpreted such course can be occurred by embolic infarct associated with a hypercoagulation state. Additionally, MRI revealing an infarction in the caudate nucleus (striatocapsular infarction) and cerebral cortex suggested an embolic infarct.

The patient was then treated with heparin, which improved her aphasia over time. Her auditory comprehension improved significantly within a few days after treatment, but her motor aphasia remained. Based on previous reports [4-14], we considered hysterectomy to be effective in preventing the recurrence of cerebral infarction. Careful discussion (among her and her family, and the medical team) was made considering that she was not postmenopausal. Finally, she decided to have surgery in light of the risk of recurrence of cerebral infarction in the future. The patient underwent a total hysterectomy on day 20. We switched heparin to rivaroxaban (15 mg/day) on day 33 for the treatment of deep vein thrombosis. Subsequent blood tests confirmed normalization of tumor markers and D-dimer, and a follow-up MRI showed no new infarcts. Her severe right upper limb paralysis remained, but her right lower limb paralysis gradually improved. She was transferred to the rehabilitation hospital on day 56. Among 16 months since the onset of cerebral infarction, she has not experienced a recurrence of infarction or thrombotic events.

## Discussion

We reviewed studies on patients with cerebral infarctions associated with uterine adenomyosis (Table 2). Patients were characterized by menstruation onset [4,5,9,11-15], high levels of CA125 [4-15] and CA19-9 [7-9,11,13-15], and D-dimer [5-15]. Among the 13 patients who did not undergo hysterectomy, four experienced recurrences of cerebral infarction under the administration of antiplatelet or anticoagulant drugs [5,9,14,15], of whom three were also receiving hormone therapy [5,9,15]. Moreover, three cases underwent a hysterectomy after the recurrence of brain infarction [9,14,15]. These three cases, together with an additional four cases, who had undergone hysterectomy at the onset of the first brain infarction, showed no recurrence of cerebral infarction after surgery [7-9,11,13-15]. There were a few cases that had co-occurrence of thrombi in other organs: brachiocephalic artery occlusion in one case [4], renal infarction in three cases [4,13,14], and splenic infarction in one case [9].

**Table 2:** A literature review of cerebral infarction associated with uterine adenomyosis CA125: cancer antigen 125; CA19-9: carbohydrate antigen 19-9; NBTE: nonbacterial thrombotic endocarditis; N/A: not applicable; GnRH: gonadotropin-releasing hormone.

Sr. No.	Age	Onset during menstruation	CA125 (U/mL)	CA19-9 (U/mL)	D-dimer (µg/mL)	NBTE	Other infarction or thrombosis	Treatment	Secondary prevention	Treatment for adenomyosis	Reference
1	45	No	159	N/A	1.1	-	Brachiocephalic artery	Heparin	Antiplatelet	GnRH agonist	[4]
2	44	N/A	-	N/A	N/A	-	Renal infarction	Heparin	Warfarin	GnRH agonist	[4]
3	50	Yes	42.6	N/A	0.57	-		Aspirin	Aspirin	GnRH agonist	[4]
4	42	Yes	1750	N/A	6	-		Antiplatelet	Antiplatelet	GnRH agonist	[5]
	Recurrence							Heparin	Warfarin	GnRH agonist	[5]
5	59	N/A	334.8	N/A	7	+		Anticoagulant	-	-	[6]
6	48	N/A	901	1791	1.9	+		Heparin	Warfarin	Hysterectomy	[7]
7	49	No	379	69.2	3.99	+		Anticoagulant	Warfarin	Hysterectomy	[8]
8	44	Yes	2115	1824	17	-	Splenic infarction	Heparin	Rivaroxaban	GnRH agonist	[9]
	Recurrence							-	-	Hysterectomy	[9]
9	42	N/A	395	N/A	1.4	-		-	Warfarin	-	[10]
10	50	N/A	143	N/A	3.7	-		-	Rivaroxaban	-	[10]
11	34	Yes	937.1	462.5	1.05	-		-	-	-	[11]
12	37	Yes	735.7	43.2	12.04	-		-	-	-	[11]
13	46	Yes	546.5	1076.6	2.34	-		-	-	Hysterectomy	[11]
14	34	Yes	937.7	N/A	27.4	-		Heparin	Antiplatelet	-	[12]
15	48	Yes	3536.2	892.1	79.3	-	Renal infarction	Mechanical thrombectomy + heparin	Edoxaban	Hysterectomy	[13]
16	47	Yes	90.3	52.3	3.8	-		Heparin	Edoxaban	-	[14]
	Recurrence						Renal infarction	Heparin	-	Hysterectomy	[14]
17	50	Yes	999	112	6.4	-		Heparin	Apixaban	GnRH agonist	[15]
	Recurrence							Heparin	-	Hysterectomy	[15]
18	46	Yes	1477	76.4	7.4	-	Deep vein thrombosis	Heparin	Edoxaban	Hysterectomy	Our case

It has been suggested that adenomyosis can cause coagulopathy [1]. In particular, cerebral infarction caused by coagulopathy due to adenomyosis seems to exhibit similarities to Trousseau’s syndrome. Evidence has shown that the pathogenesis of Trousseau’s syndrome involves the overproduction and overactivity of tissue factors, with mucinous proteins, such as CA125, themselves causing an embolus [16]. Additionally, mucin-producing tumors, such as adenocarcinomas, affect the ability of neutrophils and platelets to adhere and induce a thrombus and embolus [17]. Thus, Trousseau’s syndrome is a phenomenon that involves multiple factors, such as the overproduction and overactivity of tissue factors, the presence of mucinous proteins, and increased adhesion of neutrophils and platelets. Trousseau’s syndrome has several similarities with cerebral infarction due to adenomyosis, suggesting that coagulation disorders may occur via the same mechanism.

Our case experienced an onset of brain infarction during menstruation and markedly high levels of CA125, which was similar to those of the previous reports on the recurrence of cerebral infarction, and deep vein thrombosis, suggesting that coagulopathy may be present. Atherothrombotic cerebral infarction was also differentiated from the background of diabetes and the slowly progressive course, but when considering the association with deep vein thrombosis comorbidity from the early onset of cerebral infarction and elevated tumor markers, it was considered unlikely. In addition, screening blood tests for juvenile cerebral infarction such as hyperphospholipid antibody syndrome were also negative. The possibility of cardiogenic cerebral embolism or paradoxical cerebral embolism was not suspected more actively by the results of cardiac ultrasonography and electrocardiogram.

Although 17 cases of uterine adenomyosis with cerebral infarction have been reported, our case is the first reported case that complicated remarkable deep vein thrombosis. Based on the reported cases, hysterectomy might be the most effective approach for preventing the recurrence of brain infarction derived from adenomyosis, and our case suggests hysterectomy may be effective for both cerebral infarction and deep vein thrombosis.

## Conclusions

We herein report a case of cerebral infarction and deep vein thrombosis that was thought to have been caused by coagulopathy due to uterine adenomyosis. Both of them might be caused by a common mechanism, hypercoagulation. The mechanism by which coagulopathy developed might be similar to that for Trousseau’s syndrome, with evidence suggesting that hysterectomy may be the most effective therapy for preventing recurrence.
